# Longitudinal associations between perceptions of the neighbourhood environment and physical activity in adolescents: evidence from the Olympic Regeneration in East London (ORiEL) study

**DOI:** 10.1186/s12889-019-8003-7

**Published:** 2019-12-30

**Authors:** Nicolas Berger, Daniel Lewis, Matteo Quartagno, Edmund Njeru Njagi, Steven Cummins

**Affiliations:** 1 Population Health Innovation Lab, Department of Public Health, Environments and Society, London School of Hygiene & Tropical Medicine, 15-17 Tavistock Place, London, UK; 2 Data Science Campus, Office for National Statistics, London, UK; 3 MRC Clinical Trials Unit, University College London, London, UK; 4 Department of Medical Statistics, London School of Hygiene & Tropical Medicine, London, UK; 5 Department of Non-communicable Disease Epidemiology, London School of Hygiene & Tropical Medicine, London, UK

**Keywords:** Adolescents, East London, Environment, Neighbourhood, Perception, Physical activity, Walking

## Abstract

**Background:**

Most UK adolescents do not achieve recommended levels of physical activity. Previous studies suggested that perceptions of the neighbourhood environment could contribute to explain differences in physical activity behaviours. We aimed to examine whether five measures of perceptions – perceived bus stop proximity, traffic safety, street connectivity, enjoyment of the neighbourhood for walking/cycling, and personal safety – were longitudinally associated with common forms of physical activity, namely walking to school, walking for leisure, and a composite measure of outdoor physical activity. We further aimed to investigate the moderating role of gender.

**Methods:**

We used longitudinal data from the Olympic Regeneration in East London (ORiEL) study, a prospective cohort study. In 2012, 3106 adolescents aged 11 to 12 were recruited from 25 schools in 4 deprived boroughs of East London. Adolescents were followed-up in 2013 and 2014. The final sample includes 2260 adolescents surveyed at three occasions. We estimated logistic regression models using Generalised Estimating Equations to test the plausibility of hypotheses on the nature of the longitudinal associations (general association, cumulative effect, co-varying trajectories), adjusting for potential confounders. Item non-response was handled using multiple imputation.

**Results:**

Longitudinal analyses indicate little evidence that perceptions of the neighbourhood are important predictors of younger adolescent physical activity. There was weak evidence that greater perceived proximity to bus stops is associated with a small decrease in the probability of walking for leisure. Results also indicate that poorer perception of personal safety decreases the probability of walking for leisure. There was some indication that better perception of street connectivity is associated with more outdoor physical activity. Finally, we found very little evidence that the associations between perceptions of the neighbourhood and physical activity differed by gender.

**Conclusions:**

This study suggests that younger adolescents’ perceptions of their neighbourhood environment, and changes in these perceptions, did not consistently predict physical activity in a deprived and ethnically diverse urban population. Future studies should use situation-specific measures of the neighbourhood environment and physical activity to better capture the hypothesised processes and explore the relative roles of the objective environment, parental and adolescents’ perceptions in examining differences in types of physical activity.

## Background

Regular physical activity has been shown to prevent major non-communicable diseases and improve physical and mental health [1, 2]. Current recommendations for children and adolescents are to accumulate a minimum of 60 min of moderate to vigorous physical activity each day [3]. However, most adolescents do not achieve the recommended level of physical activity in the United Kingdom (UK) [4]. Addressing this lack of physical activity is particularly important because adolescence marks a key transition period during which life-long health behaviours start forming [5].

Amongst the multiple levels of hypothesised determinants of physical activity, proximal factors, such as exposure to the neighbourhood environment, are more amenable to modification and thus a potential target for public health interventions [6]. Younger adolescents (10–14 years) are particularly likely to be affected by the neighbourhood environment as this is where they spend the majority of their free time [7–9].

In the UK, the evidence has indicated that ethnic minorities and more deprived populations are at higher risk of physical inactivity. These populations, who may spend a greater proportion of their time in their residential neighbourhood [10], are also expected to be more affected by negative aspects of their neighbourhood social and physical environment, such as crime and disorder [11–13].

While associations between objective measures of the built environment and physical activity have been intensively studied [14], perceptions of the neighbourhood environment have been less well explored [15, 16]. Instead of considering perceptions as proxies for more objective measures, current research suggests that there are differences between these two types of environmental exposure [17, 18]. Perceptions may be affected by physical characteristics of the surroundings, but also by a variety of personal characteristics such as gender, social and cultural norms and values and socio-economic circumstances [17]. It has therefore been suggested that perceptions of the neighbourhood environment may be more proximal to health behaviour than objective measures, and mediate some of its influences [19]. Perception measures often target features of the neighbourhood that are intrinsically qualitative – such as fear of crime, aesthetics or quality of neighbourhood infrastructure (e.g. parks) – and are therefore difficult to capture using objective measures. As a result, the recent literature has indicated that objective measures and perceptions of the neighbourhood environment are complementary predictors of physical activity behaviours [17]. Whereas objective measures are more likely to capture the direct influence of neighbourhood physical characteristics, perceptions are the results of a complex interplay between the physical environments, social and intra-individual processes.

Past research has indicated that some perceptions of the neighbourhood environment are correlated with several domains of physical activity, despite the diversity of measures and approaches used. The most consistent association appears to be between perceived access to destinations and walking and other physical activity in adults [20–23], and between perceived access to recreational facilities and leisure-time physical activity in both adults and young people [24–26]. Reasonably consistent associations were also found between perceived connectivity and walking in adults [20, 26]. Other perceptions of the neighbourhood have shown mixed results and most perceptions were understudied in young people [24, 25].

Several additional gaps remain in the literature. First, there is currently little understanding as to whether younger adolescents own perceptions of their neighbourhood environment, as opposed to those of their parents, are relevant in predicting physical activity behaviours. Second, gender differences have not been systematically documented despite well-established differences in the amount and types of physical activity between boys and girls. Third, most of the literature is based on cross-sectional studies, which provide little insight as to whether physical activity might change as a result of changes in perceptions [26]. Fourth, the current literature is dominated by North American and Australian studies. More research is needed in the UK in order to corroborate results obtained in other settings and to explore potentially important contextual differences. Fifth, deprived and ethnic minority populations have been little studied, despite the fact that they are generally at greater risk of physical inactivity and are more likely to be more exposed to less supportive neighbourhood environments. Lastly, despite a growing recognition that different features of the environment affect different domains or forms of physical activity [18], few empirical studies have systematically investigated associations between features of the neighbourhood environment and domains or forms of physical activity such as walking to school, walking for leisure and leisure sport activities. As a result, the current literature still lacks robust understanding of what specific aspects of physical activity are influenced by what perceptions of the environment.

In this paper we use the Olympic Regeneration in East London (ORiEL) study to test the plausibility of alternative hypotheses on how measures of neighbourhood perceptions might influence three common forms of physical activity (walking to school, walking for leisure, and outdoor physical activity) in a deprived and ethnically diverse young adolescent population. We assessed the plausibility of three hypotheses about the nature of the longitudinal associations between perceptions of the neighbourhood environment and physical activity: do we observe a general association over time? (question 1); does the accumulation of perceptions predict physical activity at follow-up? (question 2); do trajectories of perceptions and physical activity co-vary? (question 3). We further investigate the moderating role of gender in these associations.

## Methods

### Study design and participants

We analysed data from the Olympic Regeneration in East London (ORiEL) study, a prospective cohort study aimed at assessing the health impact of urban regeneration following the London 2012 Olympic and Paralympic Games. Participants were recruited from 25 schools in four London boroughs: Tower Hamlets, Hackney, Barking and Dagenham, and Newham. The boroughs have highly ethnically diverse populations and higher levels of social, economic and environmental deprivation than the England and London average [27, 28]. Six schools per borough in Newham, Hackney and Barking & Dagenham, and seven schools in Tower Hamlets were selected using simple randomisation with refusals replaced by eligible schools from the same borough. Special-needs schools, pupil referral units and independent schools were excluded from the sampling frame. The sample consisted of both single and mixed-sex faith and non-denominational schools. Faith schools were affiliated to a range of religious denominations. Full details on study recruitment and data collection are described elsewhere [29].

The participants, in year 7 at baseline (age 11–12 years: Jan-June 2012), were first followed-up in year 8 (wave 2, age 12–13 years: Jan-June 2013) and again in year 9 (wave 3, age 13–14 years: Jan-June 2014). Timing of follow-up for each school was matched by month to reduce seasonality effects. The baseline survey comprised 3106 respondents. The final longitudinal cohort comprised 2260 adolescents who participated in all three waves, representing an overall retention rate of 73%.

### Measures

#### Perceptions of the neighbourhood environment

Adolescents were asked questions related to perceptions of their neighbourhood on selected domains, using an adapted, age-appropriate version of the ALPHA (Assessing Levels of Physical Activity and Fitness) questionnaire [30]. The ALPHA questionnaire has been used in multiple European countries and its validity and reliability assessed [30–33]. The neighbourhood was defined as an area within a 10–15 min walk from his/her house and most of the questions were specifically targeting walking and cycling behaviours. Following confirmatory factor analyses, we created summary ordinal scores to capture traffic safety and street connectivity, differentiating three types of perceptions: low support, medium support and high support of the environment [31]. Items on bus stop proximity and enjoyment of the neighbourhood for walking/cycling were used individually because we expected stronger associations between the individual items and some of the physical activity outcomes. We also used an item on personal safety from the Multi-Ethnic Study of Atherosclerosis (MESA) questionnaire [34] because it was the best proxy available for fear of crime, which is hypothesised to be associated with physical activity [35].

To answer questions about the nature of the relationships between perceptions of the environment and physical activity we additionally created longitudinal-specific exposure variables, capturing cumulative perceptions and trajectories. Cumulative exposure scores were created using the numeric values to which each response category was coded in the ordinal scores (e.g. ‘strongly disagree’ = 1, ‘slightly disagree’ = 2, …, ‘strongly agree’ = 5). For each adolescent a total score was calculated as the sum of the values across the three waves. Higher scores reflect an overall good perception of the neighbourhood environment during the study period. The same numeric values of the ordinal scores were used to compute the difference between wave 3 values and wave 1. The trajectory scores measure changes since wave 1 on a continuous scale so that positive values represents improvement of perception.

#### Physical activity outcomes

Physical activity was assessed using the Youth Physical Activity Questionnaire (Y-PAQ). Y-PAQ is a validated self-reported tool that captures the frequency and duration of a range of physical and sedentary activities over the past 7 days [36]. Three forms of physical activity expected to be differentially associated with the exposure variables were computed: walking to school, walking for leisure and outdoor physical activity [30, 35, 37, 38]. Outdoor physical activity aims to group physical activities that are mainly performed in open recreation areas such as parks, sport fields and other open spaces, which are usually located in the residential neighbourhood of the adolescents [37, 39]. It combines basketball/volleyball (with the expectation that basketball is mainly reported in an outdoor court), (roller) blading, cricket, football, rounders, rugby and roller skating. Running was not included due to reporting which reflects that the activity was likely to have been understood as ‘running around’ by adolescents and not understood as a formal sporting activity. Owing to their non-normal distributions and to the fact that no adequate transformation could be found, the three outcome variables measuring forms of physical activity were dichotomised (e.g. activity reported at least once vs. not).

#### Covariates

Potential confounders available at wave 1 and for both follow-up surveys were identified a priori from existing literature. They were included in adjusted models if there was evidence of an association with physical activity and neighbourhood perceptions. Gender; ethnicity (White British, White Mixed, Indian, Pakistani, Bangladeshi, Black Caribbean, Black African, Other); family affluence score (low, medium high); health condition (none vs. one); and free-school meal status at wave 1 were selected. Season was associated with some outcomes but in an unexpected direction (such that more physical activity is reported in winter compared to spring). As including season in the models did not change the coefficient estimates in preliminary complete case analyses, the variable was excluded from the final models.

### Statistical analyses

Prevalence of missing data for the outcomes and covariates was examined; missing values ranged from 0.0 to 18.8%. Missing data were handled using multilevel multiple imputation [40] (see Additional file 1 for details).

Unadjusted and adjusted logistic regression models were estimated using Generalised Estimating Equations (GEE) in Stata 15. GEE methods were used to account for the hierarchical structure of the data. This analytical approach is convenient because it can be used to answer research questions involving repeated measurements on the same adolescents (questions 1 and 3) or research questions involving clustering at school-level due to the study design (questions 2). The method was however restricted to two levels of analysis. In this study, GEE was preferred over generalised linear mixed models (such as multilevel logistic regression) owning to the interpretation of the parameters. Models estimated with GEE indeed have a convenient population-average interpretation of the parameters, as opposed to a subject-specific interpretation [41]. This means that the parameters of a logistic regression estimated with GEE can be interpreted in the same way as those of a traditional logistic regression.

Three types of models were fitted to answer the research questions about the nature of the longitudinal associations. Models testing for general associations obtained with pooled longitudinal models (question 1) and longitudinal models for trajectory of exposure (question 3) accounted for clustering due to repeated measurements on the same individuals. We were unable to adjust for clustering at school-level in those models. Models for cumulative exposure (question 2) presented the data as if it were cross-sectional and therefore could account for clustering at school-level.

We first fitted unadjusted models including each of the five exposure variables in turn and a physical activity outcome. Fully adjusted models were then specified, adjusting for all five exposure variables together with the potential confounders. Finally, we explored whether gender was moderator by running a series of fully adjusted models that further included an interaction term between each exposure of interest and gender, with one gender*exposure interaction at a time (i.e. one per model). Stratum-specific results were reported for *p*-values for the interactions < 0.1.

## Results

Physical activity prevalence declined over time (Table 1). The prevalence of walking to school was 77.5% at wave 1 and slightly decreased at each subsequent wave (wave 2 = 76.5%; wave 3 = 76.0%). The prevalence of walking for leisure was 40.1% at wave 1 and decreased to 34.3% at wave 2 and 29.9% at wave 3. Outdoor physical activity was highest at wave 1 (79.3%) and decreased to 76.1% at wave 2 and 70.0% at wave 3. Outdoor physical activity was higher in boys, walking for leisure higher in girls, and no gender differences was observed in walking to school (Additional file 2).

**Table 1:** Characteristics of the study participants by wave, 2012–2014 (*n* = 2260)

	Wave 1	Wave 2	Wave 3	% Missing
Exposures				
Perceived bus stop proximity				10.4
% Further away	25.5	20.7	18.8	
% 1–5 min	74.5	79.3	81.2	
Perceived traffic safety				13.4
% Low	10.5	10.4	9.9	
% Medium	31.3	35.4	34.0	
% High	58.2	54.2	56.1	
Perceived street connectivity				18.8
% Low	22.1	20.1	19.8	
% Medium	56.8	56.7	59.6	
% High	21.1	23.2	20.6	
Enjoyment of neighbourhood for walking/cycling				12.6
% Strongly/slightly disagree	23.5	25.6	25.5	
% Slightly agree	33.8	39.2	43.0	
% Strongly agree	42.7	35.2	31.5	
Feeling safe (personal safety)				15.1
% Strongly disagree	10.1	10.3	9.4	
% Slightly disagree	16.8	15.3	15.6	
% Neither agree nor disagree	24.0	23.5	22.6	
% Slightly agree	23.6	25.2	27.2	
% Strongly agree	25.6	25.8	25.3	
Outcomes				
% walking to school	77.5	76.5	76.0	4.9
% walking for leisure	40.1	34.3	29.9	10.0
% reporting outdoor physical activity	79.3	76.0	70.0	14.5
Covariates				
% Girls	43.6	–	–	0.0
Ethnicity				0.0
% White: British	16.9	–	–	
% White: Mixed	8.4	–	–	
% Asian: Indian	3.8	–	–	
% Asian: Pakistani	3.8	–	–	
% Asian: Bangladeshi	14.9	–	–	
% Black: Caribbean	4.9	–	–	
% Black: African	11.1	–	–	
% Other	36.2	–	–	
% with health condition	42.4	39.3	41.0	10.9
% receiving free school meals at wave 1	37.7	–	–	2.0
Family affluence				3.9
% Low	10.7	7.0	5.0	
% Medium	53.3	50.6	51.1	
% High	36.0	42.4	43.9	

Perceptions of the neighbourhood environments had relatively stable distributions, despite important within-person changes over time (see Additional file 3 and Additional file 4 for illustration). Perceptions of bus stop proximity slightly increased after wave 1, and the prevalence of high enjoyment of neighbourhood for walking/cycling (i.e. ‘strongly agree’) gradually decreased from 42.7 to 31.5% between wave 1 and wave 3. Low perceived street connectivity and high personal safety were higher for boys (Additional file 2). Table 1 describes the key socio-demographic characteristics of the sample. In general, the sample was ethnically diverse (only 16.9% were White British and 36.2% were classified as ‘Other’) and relatively deprived (37.7% received free school meals at wave 1; 36.0–43.9% had high family affluence).

### Walking to school

Results from the pooled model (question 1) indicates no evidence of an association between perceptions of the neighbourhood and walking to school (Table 2). Alternative hypotheses about cumulative impact (question 2) and trajectories (question 3) confirm the absence of associations with walking to school (Table 3). The inclusion of interaction terms between gender and each measure of perception of the neighbourhood indicates no evidence that gender moderates the associations.

**Table 2:** General associations of perceptions of the neighbourhood environment with walking to school across the 3 waves (*n* = 2260)

Exposure		Unadjusted			Adjusted^a^			Gender-interaction^b^
		OR	95%CI	*p*-value	OR	95%CI	*p*-value	*p*-value
Perceived bus stop proximity	Further away	1.00		0.140	1.00		0.177	0.890
	1–5 min	0.89	[0.77,1.04]		0.90	[0.78,1.05]		
Perceived traffic safety	Low	1.00		0.505	1.00		0.369	0.501
	Medium	1.11	[0.93,1.33]		1.13	[0.94,1.36]		
	High	1.10	[0.92,1.32]		1.14	[0.94,1.38]		
Perceived street connectivity	Low	1.00		0.303	1.00		0.245	0.863
	Medium	1.10	[0.95,1.27]		1.10	[0.95,1.28]		
	High	1.14	[0.96,1.36]		1.16	[0.97,1.40]		
Enjoyment of neighbourhood for walking/cycling	Strongly/slightly disagree	1.00		0.446	1.00		0.189	0.456
	Slightly agree	1.02	[0.89,1.18]		1.00	[0.86,1.17]		
	Strongly agree	0.94	[0.81,1.09]		0.89	[0.75,1.05]		
Feeling safe (personal safety)	Strongly disagree	1.00		0.770	1.00		0.700	0.841
	Slightly disagree	1.14	[0.92,1.42]		1.14	[0.91,1.42]		
	Neither agree nor disagree	1.04	[0.85,1.27]		1.02	[0.83,1.27]		
	Slightly agree	1.06	[0.86,1.31]		1.07	[0.85,1.34]		
	Strongly agree	1.08	[0.88,1.34]		1.11	[0.89,1.40]		

Results are from logistic regression models estimated with Generalised Estimating Equations to account for the dependency across repeated measurements (unstructured working correlation matrix)

^a^ Adjusted for gender, ethnicity, health condition, family affluence, free school meal status at wave 1, time and the other perception variables of the table

^b^ The adjusted model was replicated for each exposure with an additional interaction term between gender and exposure

**Table 3:** Associations of cumulative perceptions of the neighbourhood environment and trajectories of perceptions with walking to school (*n* = 2260)

	Unadjusted			Adjusted			Gender-interaction^c^
	OR	95%CI	*p*-value	OR	95%CI	*p*-value	*p*-value
Cumulative perception^a^							
Perceived bus stop proximity	0.92	[0.81,1.04]	0.171	0.91	[0.80,1.04]	0.169	0.857
Perceived traffic safety	1.01	[0.94,1.09]	0.772	1.04	[0.97,1.13]	0.278	0.938
Perceived street connectivity	1.03	[0.95,1.12]	0.514	1.04	[0.96,1.13]	0.315	0.647
Enjoyment of neighbourhood for walking/cycling	0.97	[0.90,1.04]	0.359	0.96	[0.88,1.04]	0.295	0.849
Feeling safe (personal safety)	0.98	[0.95,1.02]	0.324	0.99	[0.95,1.03]	0.699	0.867
Trajectory of perception^b^							
Perceived bus stop proximity	1.06	[0.93,1.02]	0.367	1.07	[0.94,1.23]	0.319	0.956
Perceived traffic safety	0.96	[0.88,1.04]	0.323	0.97	[0.89,1.06]	0.496	0.365
Perceived street connectivity	1.01	[0.93,1.10]	0.828	1.02	[0.93,1.11]	0.716	0.862
Enjoyment of neighbourhood for walking/cycling	0.96	[0.89,1.02]	0.196	0.96	[0.89,1.03]	0.240	0.605
Feeling safe (personal safety)	0.99	[0.91,1.01]	0.606	1.00	[0.95,1.04]	0.890	0.944

^a^ Results are from logistic regression models estimated with Generalised Estimating Equations to account for the clustering of individuals within schools (exchangeable working correlation matrix). The cumulative exposure are continuous variables constructed as the sum of scores of each exposure over the 3 waves. A higher score indicates a perception of supportive environment for the specific exposure. Adjusted models adjust for gender, ethnicity, health condition (at wave 3), family affluence (at wave 3), free school meal status at wave 1 and the other perception variables

^b^ Results are from logistic regression models estimated with Generalised Estimating Equations to account for the dependency across repeated measurements (unstructured working correlation matrix). Each exposure variable measures change since wave 1 on a continuous scale. Each unit represents an average change in exposure by one category between the baseline and the end of the study (+ 1 = improvement of the neighbourhood by one category on average). The coefficients represent the time*trajectory interaction, which assesses whether exposure trajectory is associated with different trajectory of change in the outcome. Adjusted models adjust for time, gender, ethnicity, health condition, family affluence and free school meal status at wave 1, the other perception variables, and their time*trajectory interactions

^c^ The adjusted models were replicated with the addition of interaction terms with gender

### Walking for leisure

Results from the pooled model indicate some evidence that perception of proximity to a bus stop is associated with less walking for leisure (adjusted OR = 0.89; 95% CI: 0.78–1.02) (Table 4). There is also some evidence that increased personal safety is associated with more walking for leisure (*p*-value = 0.034). In particular, adolescents who feel very unsafe (i.e. ‘strongly disagree’) had lower odds of walking for leisure compared to the other groups. The adjusted OR of slightly disagree vs. strongly disagree is 1.28 (95% CI: 1.02–1.62; p-value = 0.033) and the OR of slightly agree vs. strongly disagree is 1.31 (95% CI: 1.04–1.65; p-value = 0.020).

**Table 4:** General associations of perceptions of the neighbourhood environment with walking for leisure across the 3 waves (*n* = 2260)

Exposure		Unadjusted			Adjusted^a^			Gender-interaction^b^
		OR	95%CI	*p*-value	OR	95%CI	*p*-value	*p*-value
Perceived bus stop proximity	Further away	1.00		0.050	1.00		0.086	0.760
	1–5 min	0.88	[0.78,1.05]		0.89	[0.78,1.02]		
Perceived traffic safety	Low	1.00		0.372	1.00		0.298	0.709
	Medium	0.90	[0.75,1.08]		0.90	[0.75,1.09]		
	High	0.88	[0.74,1.05]		0.86	[0.71,1.04]		
Perceived street connectivity	Low	1.00		0.149	1.00		0.267	0.964
	Medium	1.15	[0.99,1.34]		1.13	[0.97,1.32]		
	High	1.10	[0.93,1.31]		1.09	[0.91,1.31]		
Enjoyment of neighbourhood for walking/cycling	Strongly/slightly disagree	1.00		0.360	1.00		0.534	0.353
	Slightly agree	1.02	[0.89,1.17]		1.02	[0.88,1.18]		
	Strongly agree	1.10	[0.95,1.27]		1.09	[0.92,1.29]		
Feeling safe (personal safety)	Strongly disagree	1.00		0.068	1.00		0.034	0.881
	Slightly disagree	1.28	[1.03,1.59]		1.28	[1.02,1.62]		
	Neither agree nor disagree	1.09	[0.88,1.34]		1.09	[0.87,1.36]		
	Slightly agree	1.24	[1.01,1.54]		1.31	[1.04,1.65]		
	Strongly agree	1.11	[0.90,1.38]		1.18	[0.93,1.49]		

Results are from logistic regression models estimated with Generalised Estimating Equations to account for the dependency across repeated measurements (unstructured working correlation matrix)

^a^ Adjusted for gender, ethnicity, health condition, family affluence, free school meal status at wave 1, time and the other perception variables of the table

^b^ The adjusted model was replicated for each exposure with an additional interaction term between gender and exposure

Results indicate that cumulative perceptions are not associated with walking for leisure at wave 3 (Table 5). The trajectory analysis provides some indication that increased stop proximity is associated with decreased odds of walking to school over time (*p* = 0.049).

**Table 5:** Associations of cumulative perceptions of the neighbourhood environment and trajectories of perceptions with walking for leisure (*n* = 2260)

	Unadjusted			Adjusted			Gender-interaction^c^
	OR	95%CI	*p*-value	OR	95%CI	*p*-value	*p*-value
Cumulative perception^a^							
Perceived bus stop proximity	1.00	[0.89,1.14]	0.939	0.98	[0.86,1.12]	0.798	0.844
Perceived traffic safety	1.02	[0.95,1.09]	0.596	1.04	[0.95,1.13]	0.384	0.761
Perceived street connectivity	1.04	[0.95,1.14]	0.379	1.05	[0.95,1.15]	0.354	0.471
Enjoyment of neighbourhood for walking/cycling	0.99	[0.93,1.05]	0.721	1.00	[0.92,1.08]	0.943	0.829
Feeling safe (personal safety)	0.98	[0.95,1.02]	0.319	1.00	[0.96,1.04]	0.827	0.918
Trajectory of perception^b^							
Perceived bus stop proximity	0.87	[0.76,1.05]	0.053	0.86	[0.74,1.00]	0.049	0.932
Perceived traffic safety	0.98	[0.90,1.07]	0.725	0.98	[0.90,1.08]	0.707	0.334
Perceived street connectivity	1.00	[0.91,1.10]	0.962	1.00	[0.91,1.10]	0.971	0.240
Enjoyment of neighbourhood for walking/cycling	1.02	[0.95,1.09]	0.665	1.02	[0.95,1.11]	0.583	0.260
Feeling safe (personal safety)	1.00	[0.90,1.11]	0.994	1.00	[0.95,1.05]	0.942	0.992

^a^ Results are from logistic regression models estimated with Generalised Estimating Equations to account for the clustering of individuals within schools (exchangeable working correlation matrix). The cumulative exposure are continuous variables constructed as the sum of scores of each exposure over the 3 waves. A higher score indicates a perception of supportive environment for the specific exposure. Adjusted models adjust for gender, ethnicity, health condition (at wave 3), family affluence (at wave 3), free school meal status at wave 1 and the other perception variables

^b^ Results are from logistic regression models estimated with Generalised Estimating Equations to account for the dependency across repeated measurements (unstructured working correlation matrix). Each exposure variable measures change since wave 1 on a continuous scale. Each unit represents an average change in exposure by one category between the baseline and the end of the study (+ 1 = improvement of the neighbourhood by one category on average). The coefficients represent the time*trajectory interaction, which assesses whether exposure trajectory is associated with different trajectory of change in the outcome. Adjusted models adjust for time, gender, ethnicity, health condition, family affluence and free school meal status at wave 1, the other perception variables, and their time*trajectory interactions

^c^ The adjusted models were replicated with the addition of interaction terms with gender

None of the models provide evidence that gender moderates any of these associations.

### Outdoor physical activity

The pooled model for a general association indicates weak evidence (*p*-value = 0.077) that better perception of street connectivity increases the odds of outdoor physical activity (Table 6). The odds of outdoor physical activity for those with high perception of street connectivity are 1.27 (95% CI: 1.03–1.57) times higher compared to those with low perception. Other measures of perceptions indicate no evidence of association in the adjusted model.

**Table 6:** General associations of perceptions of the neighbourhood environment with outdoor physical activity across the 3 waves (*n* = 2260)

Exposure		Unadjusted			Adjusted^a^			Gender-interaction^b^
		OR	95%CI	*p*-value	OR	95%CI	*p*-value	*p*-value
Perceived bus stop proximity	Further away	1.00		0.639	1.00		0.946	0.674
	1–5 min	0.96	[0.82,1.13]		0.99	[0.83,1.19]		
Perceived traffic safety	Low	1.00		0.490	1.00		0.182	0.012
	Medium	0.99	[0.80,1.22]		1.02	[0.82,1.29]		
	High	0.92	[0.75,1.14]		0.90	[0.71,1.14]		
Perceived street connectivity	Low	1.00		0.222	1.00		0.077	0.719
	Medium	1.05	[0.90,1.23]		1.15	[0.97,1.36]		
	High	1.18	[0.98,1.42]		1.27	[1.03,1.57]		
Enjoyment of neighbourhood for walking/cycling	Strongly/slightly disagree	1.00		0.042	1.00		0.270	0.809
	Slightly agree	0.93	[0.80,1.07]		0.95	[0.81,1.11]		
	Strongly agree	1.10	[0.94,1.29]		1.07	[0.89,1.29]		
Feeling safe (personal safety)	Strongly disagree	1.00		0.324	1.00		0.507	0.697
	Slightly disagree	1.06	[0.83,1.34]		1.12	[0.86,1.46]		
	Neither agree nor disagree	0.95	[0.75,1.19]		0.96	[0.75,1.23]		
	Slightly agree	1.06	[0.84,1.33]		1.09	[0.84,1.41]		
	Strongly agree	1.13	[0.91,1.41]		1.09	[0.85,1.39]		

Results are from logistic regression models estimated with Generalised Estimating Equations to account for the dependency across repeated measurements (unstructured working correlation matrix)

^a^ Adjusted for gender, ethnicity, health condition, family affluence, free school meal status at wave 1, time and the other perception variables of the table

^b^ The adjusted model was replicated for each exposure with an additional interaction term between gender and exposure

The inclusion of interaction terms between gender and perceptions of traffic safety indicates strong evidence that gender moderates the associations between traffic safety and outdoor physical activity (p-value = 0.012). Gender-specific results (Table 7) indicate that boys with medium or high perception of traffic safety have higher odds of outdoor physical activity compared to those with low perception of traffic safety (ORs = 1.53 (95% CI: 1.10–2.11) and 1.21 (95% CI: 0.89–1.64) respectively). In girls, the association takes the opposite direction: the odds of outdoor physical activity are lower if the perception of traffic safety is medium (OR = 0.79 (95% CI: 0.56–1.03)) or high (OR = 0.74 (95% CI: 0.52–0.96)) compared to low.

**Table 7:** Gender-specific general adjusted associations of perceptions of the neighbourhood environment with outdoor physical activity across the 3 waves (*n* = 2260)

Perceived traffic safety	OR	95%CI	*p*-value
Boys			
Low	1.00		0.002
Medium	1.53	[1.10,2.11]	
High	1.21	[0.89,1.64]	
Girls			
Low	1.00		0.147
Medium	0.79	[0.56,1.03]	
High	0.74	[0.52,0.96]	

Results are from logistic regression models estimated with Generalised Estimating Equations to account for the dependency across repeated measurements (unstructured working correlation matrix). The model is adjusted for bus stop proximity, perceived street connectivity, enjoyment of the neighbourhood for walking/cycling, personal safety, ethnicity, self-rated health, family affluence and free school meal status at wave 1

Alternative hypotheses about cumulative impact and trajectories of perceptions do not provide evidence of associations with outdoor physical activity (Table 8). There is weak evidence that trajectories of perceived proximity to a bus stop and street connectivity were differently associated with changes in outdoor physical activity for boys and for girls (*p*-values = 0.095 and 0.091, respectively). In boys, change in street connectivity was positively associated with change in outdoor physical activity (OR = 1.20 (95% CI: 1.01–1.41)); while in girls, change in perceived bus stop proximity was positively associated with change in outdoor physical activity (OR = 1.25 (95% CI: 1.99–1.57)) (Additional file 5).

**Table 8:** Associations of cumulative perceptions of the neighbourhood environment and trajectories of perceptions with outdoor physical activity (*n* = 2260)

	Unadjusted			Adjusted			Gender-interaction^c^
	OR	95%CI	*p*-value	OR	95%CI	*p*-value	*p*-value
Cumulative perception^a^							
Perceived bus stop proximity	0.95	[0.86,1.06]	0.377	0.94	[0.83,1.07]	0.331	0.900
Perceived traffic safety	1.03	[0.97,1.09]	0.332	1.00	[0.93,1.08]	0.988	0.535
Perceived street connectivity	0.99	[0.93,1.06]	0.873	1.03	[0.96,1.11]	0.398	0.816
Enjoyment of neighbourhood for walking/cycling	1.03	[0.99,1.07]	0.165	1.01	[0.95,1.08]	0.730	0.510
Feeling safe (personal safety)	1.05	[1.01,1.08]	0.006	1.02	[0.98,1.07]	0.278	0.825
Trajectory of perception^b^							
Perceived bus stop proximity	1.11	[0.95,1.29]	0.192	1.11	[0.93,1.32]	0.248	0.095
Perceived traffic safety	0.94	[0.86,1.04]	0.230	0.94	[0.84,1.04]	0.222	0.828
Perceived street connectivity	1.08	[0.98,1.18]	0.112	1.07	[0.97,1.19]	0.172	0.091
Enjoyment of neighbourhood for walking/cycling	1.02	[0.94,1.10]	0.622	1.02	[0.93,1.11]	0.737	0.527
Feeling safe (personal safety)	1.01	[0.9501.14]	0.640	1.01	[0.96,1.07]	0.734	0.956

^a^ Results are from logistic regression models estimated with Generalised Estimating Equations to account for the clustering of individuals within schools (exchangeable working correlation matrix). The cumulative exposure are continuous variables constructed as the sum of scores of each exposure over the 3 waves. A higher score indicates a perception of supportive environment for the specific exposure. Adjusted models adjust for gender, ethnicity, health condition (at wave 3), family affluence (at wave 3), free school meal status at wave 1 and the other perception variables

^b^ Results are from logistic regression models estimated with Generalised Estimating Equations to account for the dependency across repeated measurements (unstructured working correlation matrix). Each exposure variable measures change since wave 1 on a continuous scale. Each unit represents an average change in exposure by one category between the baseline and the end of the study (+ 1 = improvement of the neighbourhood by one category on average). The coefficients represent the time*trajectory interaction, which assesses whether exposure trajectory is associated with different trajectory of change in the outcome. Adjusted models adjust for time, gender, ethnicity, health condition, family affluence and free school meal status at wave 1, the other perception variables, and their time*trajectory interactions

^c^ The adjusted models were replicated with the addition of interaction terms with gender

## Discussion

In this paper we examined whether five measures of perceptions of the neighbourhood – bus stop proximity, traffic safety, street connectivity, enjoyment of the neighbourhood for walking/cycling, and personal safety – were associated with three common forms of physical activity, after controlling for individual socio-demographic characteristics. The physical activity outcomes analysed were walking to school, walking for leisure and outdoor physical activity. Analyses indicate little evidence that changes in perceptions of the neighbourhood are important predictors of younger adolescent physical activity. Specifically, walking to school was not associated with any of the five measures of perceptions. There was some evidence that greater perceived proximity to bus stops is associated with a small decrease in the probability of walking for leisure. The degree of evidence was somewhat stronger when the exposure was operationalised as a trajectory of within-adolescent change. This means that a within-individual increase in perceived proximity to a bus stop is associated with a higher probability of ceasing walking for leisure over time. Results also indicate that poorer perception of personal safety decreases the probability of walking for leisure. There was some indication that better perception of street connectivity is associated with more outdoor physical activity. Finally, despite evidence that physical activity outcomes and some perceptions differ by gender, we found very little evidence that the associations between perceptions of the neighbourhood and physical activity differed by gender.

Despite the limited number of studies on associations between perceptions of the neighbourhood and adolescent physical activity, these results provide evidence to support the argument that perceptions of the neighbourhood are not a major factor in explaining physical activity and its change over time [24, 25, 42]. Although few studies have been previously conducted in deprived multi-ethnic adolescent populations [43], these results might be surprising in light of some literature that suggests that deprived populations are expected to be more affected by some aspects of their neighbourhood such as disorder and crime neighbourhood [11].

Perceptions of proximity to bus-stops appeared to be relevant for some forms of physical activity. It was expected that perception of closer proximity to a bus stop would decrease the odds of walking to school, given that adolescents younger than 16 year old can travel by bus for free in London [44]. A negative association was observed, but it did not reach significance. However, a significant negative association was found between within-individual change in perception of bus stop proximity and change in walking for leisure. This association could indicate shift in behaviour during adolescence toward greater independent mobility and associated increased awareness of the residential neighbourhood. Previous studies have indeed indicated that the introduction of free buses in London has been associated with a reduction in the number of trips by walking, but has at the same time allowed adolescents to reach other destinations [45]. It might therefore be that an increase in bus use as adolescents get older and become more independent might be associated with the replacement of walking for leisure by other forms of activities.

Findings on the associations between crime-related safety and physical activity deserve to be discussed in light of the literature [24, 46–48]. Compared to general neighbourhood safety, it is hypothesised that fear of crime, stranger danger and personal safety – all three involving emotions and anxiety – are stronger predictors of physical activity by bringing about self or parental constraint on outdoor physical activities, including walking [49]. These associations have been confirmed in qualitative studies [50] and are expected to be particularly relevant in deprived populations, which are more at risk of crime-related safety problems [11]. Despite these theoretical expectations, we only found some evidence of an association between the MESA item on personal safety (‘I feel safe walking in my neighbourhood, day or night’) and walking for leisure. This corroborates the inconsistent results observed in previous, mostly cross-sectional, quantitative investigations [37, 48, 51–56]. Differences in the outcome measurement, exposure measurement (parents’ perceptions vs. adolescents’), study design (longitudinal vs. cross-sectional) or study setting do not appear to explain inconsistencies found in the current quantitative literature.

Two general factors might explain why few associations were observed between perceptions of the neighbourhood and physical activity. First, the measures of physical activity used are not specific to a specific location (e.g. park, neighbourhood), which can lead to an underestimation of the associations with perceptions of the neighbourhood [18, 25]. Although the study of different forms of physical activity (i.e. walking to school, walking for leisure and outdoor physical activity) is already an extension of the field compared to existing studies [25], the use of location-specific measures of physical activities is likely to increase the consistency of the results, as illustrated by some recent cross-sectional studies [37, 39]. Second, younger adolescents’ perceptions of the neighbourhood might simply not matter for physical activity. In this study, within-adolescent perceptions of the neighbourhood substantially varied over time. This could indicate that younger adolescents aged 12–14 years old do not have a well-formed perception of their environment, and that their behaviours might still depend more on their parents and their parents’ perceptions of the neighbourhood. Esteban-Cornejo et al. [37] showed that North American adolescents of a similar age tended to have different traffic-related and crime-related safety perceptions than their parents. The authors suggested that most parental perceptions were associated with some forms of physical activity, whereas adolescents’ perceptions were unrelated.

An important element of this paper has been the exploration of different ways of conceptualising the exposure-outcome association – as a general association, an association with exposure accumulation, and an overall association between trajectories. Measuring the general prediction of exposure had the greatest power to detect associations as they use both longitudinal and cross-sectional sources of information [41, 57], whereas the latter two approaches restricted the analyses to within-individual change. The findings reported here suggest that there is no evidence to support the hypothesis that the accumulation of past and current perceptions of the environment has an impact on current physical activity. We also found very limited evidence to support the hypothesis that the overall trend in perception of the environment is associated with the trend in physical activity. This might reflect the fact that within-adolescent perceptions of the neighbourhood environment measured were not consistent and fluctuated over time. The modelling strategies outlined in this paper could nevertheless be relevant for future analyses in different contexts.

### Strengths and limitations of this study

To our knowledge this is one of the first large-scale studies to longitudinally examine associations between perceptions of the neighbourhood environment with three validated measures of adolescent physical activity in UK and appropriate statistical methods to account for non-independence of observations and item non-response. The Y-PAQ questionnaire allowed for the study of three common types of physical activity, and thus explored how different aspects of physical activity were associated with perceptions of the neighbourhood environment.

A further advantage of the current study was the use of large-scale ethnically diverse sample population. The study had a high response rate (87% at wave 1) and retention rate (73%), which is consistent with best practice in other school-based cohorts [58].

This research also has limitations. Physical activity measured by the Y-PAQ is self-reported and might therefore be subject to recall and social desirability biases [59]. However, the use of objective physical activity measure was not practically possible given the size of the study. The Y-PAQ questionnaire does not have situational reference [60] and did not capture where the reported activity was taking place (e.g. garden, neighbourhood, parks). Such information would be valuable to better understand what aspects of the neighbourhood matter for specific types of activities.

As large-scale studies of ethnically diverse populations are rare in the field, especially in the UK, the ethnic diversity of the ORiEL study is a major strength. However, the super-diversity of the ORiEL sample was a limiting factor because over 200 ethnic categories were self-reported for minor groups [61], which restricted the ability to study ethnic differences in the associations between perceptions of the neighbourhood environment and physical activity. The study results presented here are therefore not generalisable as East London is a specific place with a super-diverse population with few comparators. However findings may be transferable to similar populations in urban settings.

Although the ORiEL study is one of the few large longitudinal studies to investigate the determinants of physical activity, its short period of follow-up (3 waves; 2 years) might have restricted the ability to detect longitudinal associations. Nonetheless, this study was conducted in a context of a dynamic urban setting, in which the population under study experienced accelerated physical, economic and social transformation of their residential neighbourhood, which are likely to bring about larger and faster changes in perceptions of the neighbourhood environment than would be normally observed.

As with other observational studies, we were unable to assess true causal relationships. Reverse causality could account for our findings.

## Conclusions

This study suggests that younger adolescents’ perceptions of their neighbourhood environment, and changes in these perceptions, did not consistently predict physical activity. Future studies should use situation-specific measures of the neighbourhood environment and physical activity to better capture the hypothesised processes and explore the relative roles of the objective environment, parental and adolescents’ perceptions in examining differences in types of physical activity.

## Supplementary information

**Additional file 1:** Missing data handling using multilevel multiple imputation.

**Additional file 2:** Characteristics of the study participants by gender, 2012–2014 (*n* = 2260).

**Additional file 3:** Longitudinal descriptive analysis of perceived traffic-related safety using the complete cases (*n* = 2244).

**Additional file 4:** Longitudinal descriptive analysis of perceived street connectivity using the complete cases (*n* = 2224).

**Additional file 5:** Gender-specific associations of trajectories of perceptions with outdoor physical activity (*n* = 2260).

## Data Availability

Restrictions apply to the availability of these data, which were used under license for the current study, and so are not publicly available. Data are however available from the authors upon reasonable request. Please contact SC with data requests.

## Abbreviations

ALPHA: Assessing Levels of Physical Activity and Fitness
CI: Confidence Interval
GEE: Generalised Estimating Equations
MESA: Multi-Ethnic Study of Atherosclerosis
OR: Odds Ratio
ORiEL: Olympic Regeneration in East London
UK: United Kingdom
Y-PAQ: Youth Physical Activity Questionnaire
