# Supplementary material for: Longitudinal associations between perceptions of the neighbourhood environment and physical activity in adolescents: evidence from the Olympic Regeneration in East London (ORiEL) study
Source: BMC Public Health. 2019 Dec 30;19:1760. doi: 10.1186/s12889-019-8003-7 (PMC6937816; doi:10.1186/s12889-019-8003-7)
Supplement: Supplementary file 1 — Additional file 1: Missing data handling using multilevel multiple imputation. [file 12889_2019_8003_MOESM1_ESM.docx]

**Additional file 1**

**Missing data handling using multilevel multiple imputation**

We explored both predictors of the probability of missingness and predictors of partially observed variables through logistic regression modelling. Analyses suggested that data were not missing completely at random and that the missing at random assumption was more plausible. Data were imputed using multilevel multiple imputation with the ‘jomo’ package in R, which uses a joint multivariate normal modelling approach through the Markov Chain Monte Carlo method ^1^. We imputed with 2 levels (first, adolescent; second, school) with all the outcomes and covariates as fixed effects using the data in the wide format, so that each measurement occasion was represented by a separate variable. Interaction terms between gender and perceptions of the neighbourhood environment were handled by imputing the data separately for each gender. The imputation model was chosen to be compatible with the most saturated model of interest; auxiliary variables were included both to strengthen the missing at random assumption and to recover as much information as possible ^2^. We used a ‘burn in’ period of 3,050 iterations for boys and 4,050 iterations for girls, and 500 between-imputation iterations to produce 20 imputed datasets. The Markov Chain Monte Carlo chains were examined to check for convergence.

References

1 Quartagno M, Grund S, Carpenter J. jomo: A Flexible Package for Two-level Joint Modelling Multiple Imputation. *R J* 2019; **9**: 1.

2 Carpenter JR, Kenward MG. Multiple imputation and its application. John Wiley & Sons, 2012.
