# Supplementary material for: Longitudinal associations between perceptions of the neighbourhood environment and physical activity in adolescents: evidence from the Olympic Regeneration in East London (ORiEL) study
Source: BMC Public Health. 2019 Dec 30;19:1760. doi: 10.1186/s12889-019-8003-7 (PMC6937816; doi:10.1186/s12889-019-8003-7)
Supplement: Supplementary file 2 — Additional file 2: Characteristics of the study participants by gender, 2012–2014 (n = 2260). [file 12889_2019_8003_MOESM2_ESM.docx]

**Additional file 2**

Table - Characteristics of the study participants by gender, 2012-2014 (n=2,260)

|  | **Boys** | **Girls** |
| --- | --- | --- |
| ***Exposures*** |  |  |
| Perceived bus stop proximity |  |  |
| *% Further away* | 21.6 | 21.7 |
| *% 1-5 minutes* | 78.4 | 78.3 |
| Perceived traffic safety |  |  |
| *% Low* | 10.0 | 10.7 |
| *% Medium* | 33.5 | 33.7 |
| *% High* | 56.5 | 55.7 |
| Perceived street connectivity |  |  |
| *% Low* | 22.9 | 17.8 |
| *% Medium* | 55.8 | 60.1 |
| *% High* | 21.3 | 22.1 |
| Enjoyment of neighbourhood for walking/cycling |  |  |
| *% Strongly/slightly disagree* | 25.8 | 23.8 |
| *% Slightly agree* | 37.5 | 40.1 |
| *% Strongly agree* | 36.7 | 36.2 |
| Feeling safe (personal safety) |  |  |
| *% Strongly disagree* | 10.2 | 9.6 |
| *% Slightly disagree* | 14.6 | 17.6 |
| *% Neither agree nor disagree* | 22.3 | 24.7 |
| *% Slightly agree* | 24.7 | 26.2 |
| *% Strongly agree* | 28.3 | 22.0 |
| ***Outcomes*** |  |  |
| % walking to school | 76.2 | 77.2 |
| % walking for leisure | 30.1 | 40.7 |
| % reporting outdoor physical activity | 86.8 | 60.0 |
