# Supplementary material for: Longitudinal associations between perceptions of the neighbourhood environment and physical activity in adolescents: evidence from the Olympic Regeneration in East London (ORiEL) study
Source: BMC Public Health. 2019 Dec 30;19:1760. doi: 10.1186/s12889-019-8003-7 (PMC6937816; doi:10.1186/s12889-019-8003-7)
Supplement: Supplementary file 3 — Additional file 3: Longitudinal descriptive analysis of perceived traffic-related safety using the complete cases (n = 2244). [file 12889_2019_8003_MOESM3_ESM.docx]

**Additional file 3**

Table - Longitudinal descriptive analysis of perceived traffic-related safety using the complete cases (n=2,244)

|  | **Overall** | | **Between**¹ | | **Within**² |
| --- | --- | --- | --- | --- | --- |
|  | **Freq.** | **Percent** | **Freq.** | **Percent** | **Percent** |
| Low | 599 | 10.2 | 493 | 22.0 | 46.4 |
| Medium | 1970 | 33.6 | 1358 | 60.5 | 56.2 |
| High | 3300 | 56.2 | 1772 | 79.0 | 70.6 |
| Total | 5869 | 100.0 | 3623 | 161.5 | 61.9^3^ |

Note: results are from complete case analysis and only valid under the missing completely at random assumption

¹ Number/proportion of individuals who were ever assigned to the category across all waves.

² Conditional on an individual being ever assigned to a category in any of the waves, proportion of his/her other observations that are also of the same category.

^3^ Normalized between weighted average of the ‘within percents’ (summarises the stability of the variable).
