# Supplementary material for: Longitudinal associations between perceptions of the neighbourhood environment and physical activity in adolescents: evidence from the Olympic Regeneration in East London (ORiEL) study
Source: BMC Public Health. 2019 Dec 30;19:1760. doi: 10.1186/s12889-019-8003-7 (PMC6937816; doi:10.1186/s12889-019-8003-7)
Supplement: Supplementary file 4 — Additional file 4: Longitudinal descriptive analysis of perceived street connectivity using the complete cases (n = 2224). [file 12889_2019_8003_MOESM4_ESM.docx]

**Additional file 4**

Table - Longitudinal descriptive analysis of perceived street connectivity using the complete cases (n=2,224)

|  | **Overall** | | **Between**¹ | | **Within**² |
| --- | --- | --- | --- | --- | --- |
|  | **Freq.** | **Percent** | **Freq.** | **Percent** | **Percent** |
| Low | 1121 | 20.4 | 835 | 37.5 | 54.2 |
| Medium | 3186 | 57.9 | 1836 | 82.6 | 70.3 |
| High | 1198 | 21.8 | 863 | 38.8 | 55.7 |
| Total | 5505 | 100.0 | 3534 | 158.9 | 62.9^3^ |

Note: results are from complete case analysis and only valid under the missing completely at random assumption

¹ Number/proportion of individuals who were ever assigned to the category across the waves.

² Conditional on an individual being ever assigned to a category in any of the waves, proportion of his/her other observations that are also of the same category.

^3^ Normalized between weighted average of the ‘within percents’ (summarises the stability of the variable).
