# Supplementary material for: Longitudinal associations between perceptions of the neighbourhood environment and physical activity in adolescents: evidence from the Olympic Regeneration in East London (ORiEL) study
Source: BMC Public Health. 2019 Dec 30;19:1760. doi: 10.1186/s12889-019-8003-7 (PMC6937816; doi:10.1186/s12889-019-8003-7)
Supplement: Supplementary file 5 — Additional file 5: Gender-specific associations of trajectories of perceptions with outdoor physical activity (n = 2260). [file 12889_2019_8003_MOESM5_ESM.docx]

**Additional file 5**

Table - Gender-specific associations of trajectories of perceptions with outdoor physical activity (n=2,260)

| **Trajectory of perception** | **OR** | **95%CI** | **p-value** |
| --- | --- | --- | --- |
| **Boys** |  |  |  |
| Perceived bus stop proximity | 0.92 | [0.92,1.20] | 0.554 |
| Perceived traffic safety | 0.95 | [0.95,1.12] | 0.530 |
| Perceived street connectivity | 1.20 | [1.01,1.41] | 0.034 |
| Enjoyment of neighbourhood for walking/cycling | 1.04 | [0.90,1.19] | 0.624 |
| Feeling safe (personal safety) | 1.00 | [0.93,1.09] | 0.903 |
| **Girls** |  |  |  |
| Perceived bus stop proximity | 1.25 | [0.99,1.57] | 0.055 |
| Perceived traffic safety | 0.92 | [0.81,1.06] | 0.253 |
| Perceived street connectivity | 1.00 | [0.87,1.15] | 0.962 |
| Enjoyment of neighbourhood for walking/cycling | 1.00 | [0.89,1.12] | 0.973 |
| Feeling safe (personal safety) | 1.02 | [0.95,1.09] | 0.643 |

Results are from logistic regression models estimated with Generalised Estimating Equations to account for the dependency across repeated measurements (unstructured working correlation matrix). Each exposure variable measures change since wave 1 on a continuous scale. Each unit represents an average change in exposure by one category between the baseline and the end of the study (+1 = improvement of the neighbourhood by one category on average). The coefficients represent the time*trajectory interaction, which assesses whether exposure trajectory is associated with different trajectory of change in the outcome. Models adjust for time, ethnicity, health condition, family affluence and free school meal status at wave 1, the other perception variables, and their time*trajectory interactions.
